# Saline Environments as a Source of Potential Quorum Sensing Disruptors to Control Bacterial Infections: A Review

**DOI:** 10.3390/md17030191

**Published:** 2019-03-25

**Authors:** Marta Torres, Yves Dessaux, Inmaculada Llamas

**Affiliations:** 1 Department of Microbiology, Faculty of Pharmacy, University of Granada, 18071 Granada, Spain; mtorres@ugr.es; 2 Institute of Biotechnology, Biomedical Research Center (CIBM), University of Granada, 18100 Granada, Spain; 3 Institute for Integrative Biology of the Cell (I2BC), CEA/CNRS/University Paris-Sud, University Paris-Saclay, 91198 Gif-sur-Yvette, France

**Keywords:** quorum sensing, QSI, quorum quenching, QQ, marine habitat, saline environment, hypersaline habitat, marine pathogens, plant pathogens, *Vibrio*

## Abstract

Saline environments, such as marine and hypersaline habitats, are widely distributed around the world. They include sea waters, saline lakes, solar salterns, or hypersaline soils. The bacteria that live in these habitats produce and develop unique bioactive molecules and physiological pathways to cope with the stress conditions generated by these environments. They have been described to produce compounds with properties that differ from those found in non-saline habitats. In the last decades, the ability to disrupt quorum-sensing (QS) intercellular communication systems has been identified in many marine organisms, including bacteria. The two main mechanisms of QS interference, i.e., quorum sensing inhibition (QSI) and quorum quenching (QQ), appear to be a more frequent phenomenon in marine aquatic environments than in soils. However, data concerning bacteria from hypersaline habitats is scarce. Salt-tolerant QSI compounds and QQ enzymes may be of interest to interfere with QS-regulated bacterial functions, including virulence, in sectors such as aquaculture or agriculture where salinity is a serious environmental issue. This review provides a global overview of the main works related to QS interruption in saline environments as well as the derived biotechnological applications.

## 1. Introduction

Many bacterial species have developed sophisticated cell concentration-dependent gene expression mechanisms. These are collectively called quorum sensing (QS), a term that was first introduced by Fuqua et al. in 1994 [1]. This phenomenon involves the synthesis, release and detection of signal molecules known as autoinducers, the concentration of which mirrors that of the bacterial population. Once a threshold signal concentration, i.e., a threshold bacterial concentration is reached, the presence of the signal is perceived by the emitting bacteria which in turn induce the QS-regulated biological response in a synchronous way (reviews: [2,3,4]). The first instance of QS-regulation was found in the *Vibrionaceae Photobacterium fischeri* (now *Aliivibrio fischeri*; [5]), where it regulates light emission (bioluminescence) by the bacteria in dedicated organs of marine animals [6,7]. The biological role of this symbiotic interaction is not fully understood, though it has been proposed that light production may attract some marine organisms including zooplankton ([8], review: [9]).

Multiple QS signals have been identified in bacteria. The most common ones are *N*-acylhomoserine lactones (AHLs) produced by numerous *Proteobacteria*; oligopeptides produced by *Firmicutes,* and furanosylborate diester (AI-2) produced by both *Proteobacteria* and *Firmicutes* (reviews: [2,3,4,10,11,12]). Other signals, such as 3-hydroxypalmitate (3OH-PAME; [13]), diketopiperazines (DKP; [14]), quinolones (PQS; [15]), diffusible signal factors (DSF; [16]), or resorcinol derivatives [17] have been detected in a limited number of proteobacterial species.

QS is ubiquitous in the bacterial world. It regulates different cellular functions that generally permit the adaptation of the bacteria to its environment, most often by gaining a better access to resources. For instance, QS-regulated functions include the production of antibiotics that allow the emitting bacteria to outcompete other microbes, or exoenzymes and toxins that permit bacteria to take advantage of the metabolites and tissues of other organisms that they parasite. QS-regulated functions also include the production of exopolysaccharides, the control of swarming motility or biofilm formation, the conjugal transfer of plasmids, etc. (a nonlimitative list; reviews: [2,3,4,12,18,19]). In plant and animal pathogens, some of the above QS-regulated functions are therefore determinants of the bacterial virulence or virulence-associated traits.

## 2. Quorum Sensing in Bacteria of Aquacultural Importance

The genera *Vibrio*, *Edwardsiella*, *Aeromonas*, *Pseudomonas*, and *Yersinia* encompass species that are pathogens of marine organisms [20,21,22,23,24]. Numerous studies have focused on Vibrio species that are ubiquitous in marine and estuarine ecosystems, including aquaculture farms. Some of these species, such as *V. harveyi*, *V. campbellii*, or *V. alginolyticus* are the main causative agents of diseases in marine animals that generate a high mortality rate worldwide [25,26,27,28,29,30].

**Figure 1:**
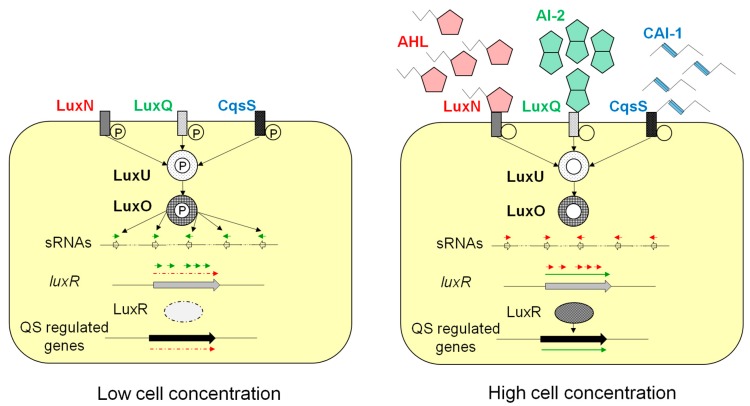
The three parallel quorum-sensing (QS) signaling pathways of *Vibrio harveyi*. The three types of QS signals (AHL, AI-2, and CAI-1) as well as the regulatory process are described below in the text. Legends: sRNAs stands for bacterial small RNAs. The circles linked to LuxN, LuxQ, CqsS, LuxU, and LuxO indicate the phosphorylation status of the proteins (P, phosphorylated; no letter, dephosphorylated). Bold arrows symbolize genes; thin arrows represent RNAs, either sRNAs or mRNAs. Red lines indicate nontranscribed RNAs; green lines, transcribed RNAs. Figure is adapted from [48].

In *Vibrio*, QS depends on at least three major signal classes: AHLs [7], AI-2 (4,5-dihydroxy-2,3-pentanedione and its boron-containing derivatives; [31,32]), and 3-hydroxytridecan-4-one (or CAI-1), the latter compound being a key regulator of pathogenicity in *V. cholerae* [33,34]. In *V. harveyi* and *V. campbellii*, the three QS signals can also be produced [10] but in these species CAI-1 slightly differs from that of *V. cholerae* as being (*Z*)-3-aminoundec-2-en-4-one [35]. This triple signalization pathway involves three different sensing systems (LuxN, Lux Q, and CqsS). Schematically (Figure 1), each of them consists of a membrane-bound histidine-kinase sensor protein that, in the absence of QS signal, activates by phosphorylation via the phosphorelay protein LuxU, the common response receptor LuxO (Figure 1, left panel). LuxO activates the transcription of sRNAs that mostly target the mRNA resulting from the transcription of *luxR*. LuxR is the main QS regulator of the transcription of QS-regulated genes. In the presence of the QS signals (Figure 1, right panel), the histidine kinases become phosphatases, a feature that eventually leads to the dephosphorylation of LuxO thus authorizing the production of LuxR and the expression of the QS-regulated genes (reviews: [10,36,37]). In *Vibrio* spp., QS-regulated genes encode the synthesis of biofilm, exoenzymes, and pigments [22,24,38,39,40,41,42,43], some being, as indicated earlier, virulence factors. For instance, the QS-controlled traits in *Vibrio campbellii* include the synthesis of siderophores that efficiently chelate iron, and that of metalloprotease, and chitinase A that can degrade the tissue of the host [30,44,45]. In *V. anguillarum* QS controls the production of metalloprotease, siderophore and biofilm [38,46] while in *V. owensii*, *V. mediterranei*, and *V. corallilyticus* QS regulates the production of exoenzymes and the swarming ability (Table 1) [47].

## 3. Inhibition of Quorum Sensing Mechanisms

In the last decades (see for instance: [49,50,51,52,53]), research has focused on one promising strategy based on the inhibition of the expression of virulence genes that are regulated by QS mechanisms (reviews: [54,55]) in proteobacteria of marine and agricultural importance (Table 1). This strategy has been termed antivirulence (reviews: [56,57]) since it aims at “disarming” the pathogens rather than killing them, thereby preventing disease induction in their host. Given that QS-regulated genes are generally not essential for the bacteria, antivirulence approaches apply less selective pressure on the pathogens and attenuate bacterial infections without decreasing growth, in contrast to antibiotics (review: [58]). Therefore, in principle, the inhibition of the cell-to-cell communication system would not lead to the development of resistance in bacteria. However, several authors have suggested that some bacteria could originate resistance mechanisms to compounds that interfere with QS [59,60,61,62,63]. This could be achieved by enhanced effluxes of signal molecules or modifications on the receptor-binding site of the LuxR-type regulator, or through the generation of spontaneous mutants that stop controlling their virulence via signal molecules and become resistant to QS interference [64,65]. However, even if resistances might happen, some authors suggested that this does not imply that it will spread [66]. Moreover, the resistance rates could be much lower than what has been observed for conventional antimicrobials (reviews: [66,67,68,69]).

**Figure 2:**
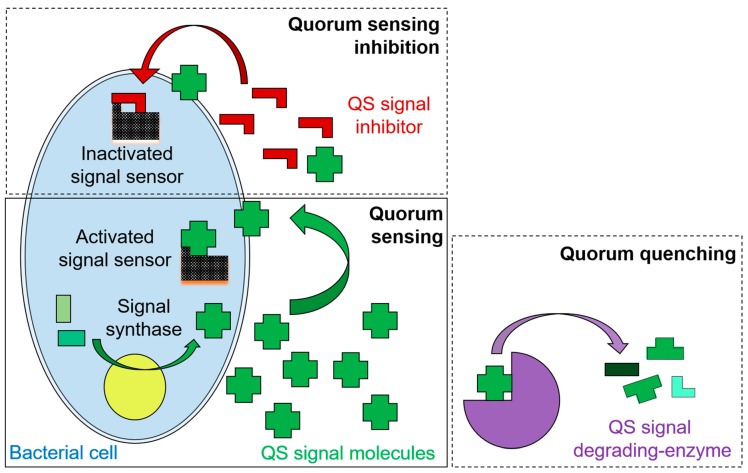
Schematic representation of a quorum sensing (QS) system and its interruption mechanisms: quorum quenching (QQ) and QS inhibition (QSI). The QS signals (green crosses) are synthesized by a synthase from the metabolic pool of the bacterial cell. They diffuse out of the cell and their presence is sensed by a bacterial sensor protein once a threshold cell, hence signal concentration, is reached (lower left panel). QS signals can however be degraded by enzymatic activity (lower right panel), preventing their detection by the bacterial cells. The presence of QS inhibitors (red L-shape figures, upper left panel) inactivate the sensors, hindering the detection of the QS signals. Both mechanisms (QQ and QSI) lead to a reduced or abolished expression of QS regulated genes.

Many organisms, including bacteria [70,71,72,73,74], yeasts [75,76,77], fungi [78,79], and marine and terrestrial plants [80,81,82,83] and animals [84,85,86] have developed the ability to disrupt AHL-based QS systems through various mechanisms (reviews: [54,55,87,88]). The first mechanism described is based on the production of molecules that act as antagonists of signals and interfere with the detection of signal molecules by their cognate transcriptional regulator (Figure 2). These molecules were termed quorum sensing inhibitors (QSIs; [80,89]). Another mechanism, known as quorum quenching (QQ; [72]), consists of the enzymatic inactivation of AHL signal molecules that abolishes bacterial QS-regulated functions (Figure 2). Nowadays, three main groups of QQ enzymes have been described based on the involved enzymatic activity. These are: (i), the AHL acylases [90] that catalyze the hydrolytic cleavage of an amide bond between the acyl chain and the homoserine lactone ring; (ii), the AHL lactonases [71] that open the lactone ring in the AHL molecule to form *N*-acylhomoserine as a product; and (iii), the AHL oxidoreductases [91] that modify the AHLs by oxidizing or reducing the acyl chain without degrading the compound (reviews: [55,92]). The biological roles of QSI production and QQ enzymes are multiple: they range from the fine tuning of QS regulated function to resistance to antimicrobial compounds, and from the recycling of QS signals to the establishment of sophisticated “decision mechanisms” (review: [55]). 

Various technical approaches were used to identify QSIs and QQ organisms and compounds that interfere with AHL signaling. Mass screenings of chemical or natural compounds libraries were instrumental to the identification of several QSIs (reviews: [93,94]), such as hordenine (*N,N*-dimethyltyramine) or the human hormone estrone and its structural relatives estriol and estradiol [95]. The primary structures of the molecules are not closely related to that of AHLs, but their spatial structures bear sufficient similarity to allow their recognition by LuxR-like regulators. A tetrazole with a 12-carbon alkyl tail, as well as *N*-nonyl-3-oxo-3-phenyl-propionamide [96] and several other AHL structural analogues [97] were also characterized in the same way. In plants, QSIs were identified either serendipitously [80] or by random tests in plants [83,98], including medicinal plants [99,100]. Drug design strategies such as protein ligand docking have also been implemented to generate molecules with putative or existing QSIs activity [101,102]. On the other hand, QQ microorganisms were identified mostly via targeted approaches. These later were based on the ability of organisms to degrade AHL signals in culture followed by the identification of the enzymatic activity [70,91,103,104,105,106]. Considering that a large part of the microbiome of natural and complex environment such as soil is still uncultivable, several authors successfully developed metagenomics strategies to identify genes encoding novel enzymes with AHL-degradation ability [97,107,108,109,110,111,112,113,114,115].

Most of the interference work targeted AHL signalization. However, several studies (review: [94]) also aimed at finding QSIs or QQ enzymes/organisms interfering with signals other than AHLs, as exemplified by the isolation of microorganisms that degrade 3-OH-PAME [116], PQS [117], and DSF [118]. Recently, an AI-2 degrading enzyme was identified via a metagenomic approach [113]. Another one was detected in an *Acinetobacter lactucae* strain isolated from activated sludge, and found to reduce biofouling in a membrane bioreactor [119]. A more comprehensive examination of the studies that aimed at AI-2 signaling can be read below in the section “Interference in marine environments”.

## 4. Saline Environments as an Important Source of Bioactive Molecules

The categories proposed by Kushner and Kamekura [196] are the most accepted by scientists when classifying microorganisms on the basis of their optimal growth rates at different salinities. Thus, microbes fall into the four following categories: extreme halophiles, which grow best in media with 15–30% *w*/*v* NaCl (2.5–5.2 M); moderate halophiles, that grow optimally in media containing 3 to 15% *w*/*v* NaCl (0.5–2.5 M); slight halophiles, that include most marine microorganisms and grow optimally in media with 1–3% *w*/*v* NaCl (0.2–0.5 M); and non-halophilic, with optimal growth in media with less than 1% *w*/*v* NaCl (0.2 M). Non-halophilic microorganisms that are able to tolerate (but do not require) high concentrations of salts are called halotolerant [196].

Saline habitats are widely distributed around the world and are represented by marine environments, saline and hypersaline lakes, solar salterns or hypersaline soils (>0.2% *w*/*v* salts), amongst others. Microorganisms that inhabit those environments are mainly halophiles, although a high amount of halotolerant organisms are also present. All of these microorganisms are adapted to grow in the presence of a high ionic content (mainly NaCl) and often to withstand other environmental stress factors such as low oxygen availability, alkaline pH values, low or high temperatures, presence of toxic compounds, etc. (reviews: [197,198]).

These specific physiochemical characteristics of saline environments may induce halophiles to synthesize unique molecules and physiological pathways to cope with the stress conditions that characterize these habitats. In fact, halophiles have been reported to produce bioactive molecules with properties that differ from those found in non-saline habitats (reviews: [199,200,201,202]). Indeed, hypersaline environments have demonstrated to be a valuable source of microorganisms that produce a number of novel compounds such as exopolysaccharides [203,204] and enzymes, such as alpha-amylases [205], endoglucanases [206], or lipases [207] that exhibit unique properties and promising perspectives for biotechnological exploitation.

In the same way as saline and hypersaline environments, the marine environment, based on its huge microbial biodiversity, is also considered as an important resource of novel bioactive compounds, including secondary metabolites used for pharmaceutical and biotechnological applications (reviews: [202,208,209]) and anti-QS substances (reviews: [114,202,210,211]) amongst other molecules.

## 5. Quorum Sensing Interference in Marine Environments

All the above data demonstrate that the ability to disrupt QS systems by different mechanisms occur in many organisms. Possibly, these phenomena could be more frequent in the marine environment than in the soil. In a study performed in bare soil and in a tobacco rhizosphere, the percentage of AHL-degrading bacteria was ca. 2 to 3% [212]. Similar ratios of QS-interfering bacteria were reported for a set of soil bacteria (5%; [70]) and bacterial isolates from a wheat rhizosphere (7%; [213]). This percentage reached 14% for dense microbial communities from marine surfaces and 28% for strains from surface oceanic samples [214]; it increased up to 84% in bacterial strains isolated from ocean at 2000 m depth [215]. Interestingly, the proportion of AHL degraders dropped as did the salinity of the water. In estuarine water (with less salt concentration than seawater), such proportion was found to be as low as 2% [111], a value comparable to that found in soil environments.

Mechanisms of QS interference, QSI and QQ, have been investigated in numerous marine organisms (reviews: [216,217,218]): micro-algae [219], macro-algae [80], invertebrates [220], fungi [221], and marine bacteria [214]. With respect to microorganisms, numerous data on QSI and QQ have been obtained from marine bacterial strains isolated from specific habitats such as aquaculture tank seawater [104,106], sediments [214,222], sponges [220,223,224], cnidarians [105], seagrass [225], and marine algae [226]. Some authors have also studied the occurrence of QQ and QSI in metagenomes obtained from diverse seawater samples from different depths and sampling places [111,114,215].

**Figure 3:**
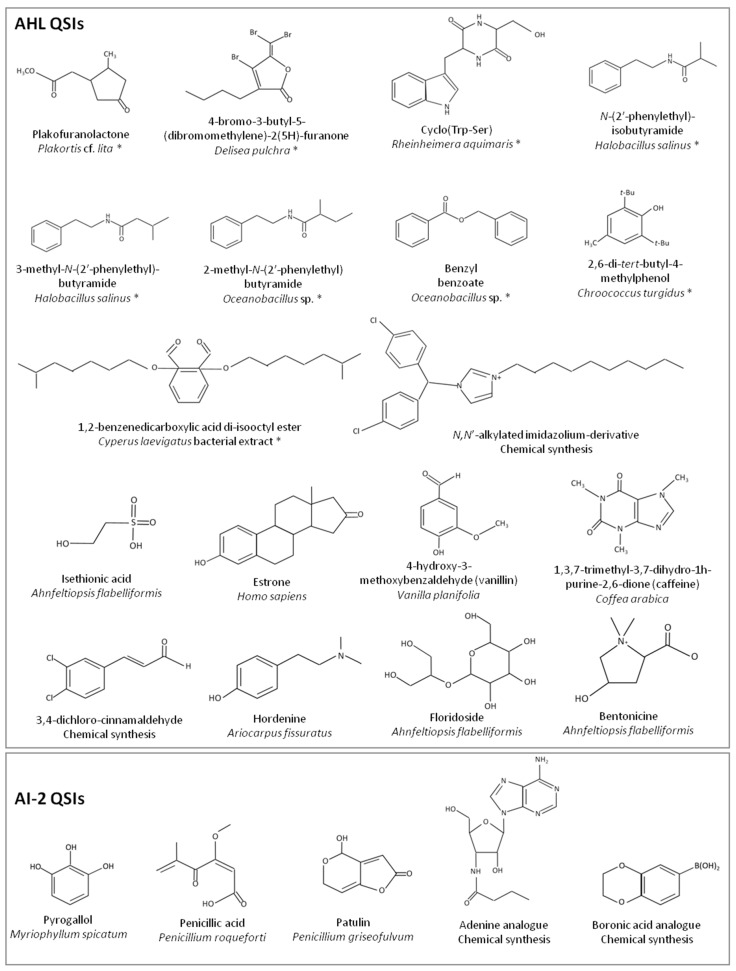
Chemical structures of some quorum sensing inhibitors (QSIs) and their origin. QSI compounds of marine origin are marked with an asterisk.

QSI occurrence was first described in the red marine alga *Delisea pulchra* that produces halogenated furanones (Figure 3) which interfere with AHL signaling and protect both shrimp and fish from vibriosis [80,136]. Since then, other furanones have been identified in marine organisms, such as plakofuranolactones which were isolated in the marine sponge *Plakortis* cf. *lita* [224]. Furanones, however, may exhibit some toxicity towards some marine organisms [140]. As a consequence, efforts were made to develop less toxic furanone derivatives retaining QSI activity [227]. QSIs of AHL molecules have been described also in the marine bacteria *Rhizobium* sp. [228], *Halobacillus salinus* [210], *Oceanobacillus* sp. [229], *Rheinheimera aquimaris* [230], and *Streptomyces* sp. [231]. Recently, 2,6-di-*tert*-butyl-4-methylphenol, a novel QSI compound isolated from the marine cyanobacteria *Chroococcus turgidus*, proved to be very effective for the control of the virulence-associated traits of *Vibrio* spp. [144] (Figure 3). Interestingly, some AHLs could also be regarded as QSIs in some specific marine systems, such as the Mediterranean sea strain *Pseudoalteromonas ulvae* [232].

QSIs were also searched for the interference of AI-2 signal communication (Figure 3). Patulin and penicillic acid, which are known as QSIs, were successfully tested on AI-2 signaling in *Halomonas pacifica* and *Marinobacter hydrocarbonoclasticus* [233]. Screening based on classical methods, e.g., bioluminescence inhibition of *Vibrio harveyi*, was used also to identify QSIs such as pyrogallol and boronic acids [145,234]. Metagenomic library screening led to the identification of adenine analogues which affect biofilm formation, decrease pigment and protease production in *V. anguillarum* and protect *Artemia* sp. from *V. harveyi*-induced mortality [235]. Drug design approaches were also implemented to identify putative AI-2 QSIs [236,237] while computer-assisted docking experiments permitted the identification of seven polycyclic compounds that drastically reduce bioluminescence in *V. harveyi* without originating cell toxicity [238,239,240].

The second antivirulence mechanism, i.e., QQ, appears to be an important process in the seawater [111]. Although QQ of AI-2-type molecules has recently been reported [113], most studies have focused on the degradation of AHLs. Indeed, QQ enzymes having AHL signals as substrates have been described in many marine species, such as *Alteromonas stellipolaris, A. genovensis, Pseudoalteromonas paragorgicola, P. tetraodonis, P. carrageenovora, P. atlantica, P. distincta* [104]; *P. flavipulchra* [241]; *A. marina, Thalassomonas agariperforans, Paracoccus homiensis* [106]; *Muricauda olearia* [242]; *Tenacibaculum maritimum* [243]; *Roseovarius aestuarii, Rhodococcus erythropolis, Salinicola salarius* [214]; *Ruegeria mobilis* [244]; *Stenotrophomonas maltophilia* [105]; *Maribacter ulvicola, Olleya marilimosa* [111]; *Planococcus* sp. [245] and *Bacillus* sp. [246].

Genes encoding AHL degradation enzymes are also abundant in marine metagenomic collections. Interestingly, searches for QQ enzymes in such collections revealed that acylases might be more abundant than lactonases [111,215], in agreement with the results obtained for cultivable bacteria. For instance, acylases have been described in *Alteromonas stellipolaris* [104], *Pseudomonas flavipulchra* [241], *Shewanella sp.* [247]*, Oceanobacillus sp.* [214]*, Stenotrophomonas maltophilia* [105] and *Anabaena* sp. [248]. On the other hand, lactonases have been identified only in some species such as *Ruegeria mobilis* [244], *Muricauda olearia* [242], *Planococcus* sp. [245] and *Tenacibaculum* sp. [249]. This comes in contrast with the situation in terrestrial environments, where AHL lactonases were more frequently isolated. Soils are generally less alkaline than seawater, the average pH of which being 8.2. At this pH value, AHLs undergo a moderate chemical lactonolysis [83,250,251] but whether this can be related to the more frequent detection of acylases activity in marine samples remains unclear.

## 6. Quorum Sensing Interference in Saline and Hypersaline Environments

Although QS inhibition has proved to be a frequent mechanism in marine aquatic environments, little is known about this phenomenon in saline and hypersaline habitats. However, a growing interest exists in the identification of novel bioactive compounds, enzymes and bacteria from extreme environments, including QQ enzymes [252], since they generally have characteristics and phenotypes—and, therefore, biotechnological applications—that differ from those retrieved in bacteria isolated from less harsh habitats (reviews: [253,254]).

The studies related to QS in saline and hypersaline habitats are also scare. The first report of QS communication systems in halophilic bacteria was conducted by Llamas et al. in 2005 [255], who described the AHL synthesis in the exopolysaccharide-producing species of *Halomonas* isolated from hypersaline soils in Spain and Morocco. Afterwards, AHL production has also been reported in 43 additional bacterial species belonging to the family *Halomonadaceae*, as well as the identification and characterization of the QS gene system *hanI*/*hanR* [256]. However, the role of QS in these bacteria has not yet been elucidated, although recently, it has been suggested that it could be related to exopolysaccharide production in the species *Halomonas smyrnensis* [257]. Regarding the other types of QS signal molecules, AI-2 production has been described in the halophilic bacteria *Halobacillus halophilus* [258], and production of DKP-type molecules has been characterized in the extremely halophilic archaeon *Haloterrigena hispanica* [259,260,261].

In relation with QS inhibition, several QSI compounds have been identified in hypersaline cyanobacterial mat in Oman [262]. More recently, the QSI compound 1,2-benzenedicarboxylic acid di-isooctyl ester that is active on the inhibition of AHL signaling in *Pseudomonas aeruginosa*, has been characterized in extracts of the bacteria isolated from the root system of smooth flatsedge (*Cyperus laevigatus*) growing in a wet saline coastal soil in India [263]. Regarding QQ enzymes, a novel AHL lactonase was identified in a metagenomic library constructed from a hypersaline soil in Spain [115]. Its expression on three aquaculture-related pathogenic *Vibrio* spp. reduce their virulence in brine shrimps (*Artemia salina*) and Manila clams (*Venerupis philippinarum*) [47]. This overall limited information can be explained by the difficulty to study QS and QQ in halophilic bacteria, since their salt requirements can inhibit the biosensors used for the detection of AHLs [255].

## 7. Applications in Aquaculture and Other Industries

To date, bacterial diseases are an important cause of mortality, causing considerable economic losses in commercial aquaculture and agriculture (reviews: [264,265,266]). Classically, antibiotics have been used in many countries to prevent and control bacteria outbreaks. However, resistances are rapidly spreading, posing a substantial problem [267,268,269,270]. Since the use of antibiotics for disease treatments and as growth promoters have been prohibited in Europe and tightly regulated in other countries, global efforts are needed in order to explore novel strategies to control bacterial pathogens and to overcome the disadvantages of antibiotics.

QS inhibition mechanisms have been reported to boast numerous biotechnological applications, which have become of great interest as alternative to other treatments. In the last decades, QQ and QSI approaches have been tested in aquaculture, agriculture, wastewater treatment, medicine and food packaging, amongst others, as reflected by the increasing number of patents within the field (reviews: [88,271,272]).

In the aquaculture sector, different studies have proved the potential value of QQ to fight bacterial infections by incorporating the AHL-degrading bacteria or QQ enzymes in the rearing water or by bioencapsulating them in the feed stock [47,104,125,133,273,274,275]. Here also, several patent applications have been registered (reviews: [88,271,272]). To date, the use of AHL-degrading marine bacteria and their purified QQ enzymes has proved to be successful in reducing or eliminating the virulence of pathogenic bacteria against fish, crustaceans, mollusks, and corals. For instance, cultures from the intestinal tract of healthy shrimp and fish enriched in QQ enzymes increase the survival rate of turbot larvae (*Scophthalmus maximus*) [275] and of giant freshwater prawns (*Macrobrachium rosenbergii*) [274]. Another example is the addition of an AHL-degrading *Alteromonas stellipolaris* strain to the rearing water, which reduces the virulence of *Vibrio mediterranei* upon the coral *Oculina patagonica* [104], or the protection of the fish *Danio rerio* and *Carassius auratus* from *Aeromonas hydrophila* infection by the addition of an AHL-degrading *Bacillus* sp. strain [124,125,273]. In the same way, the use of the purified QQ enzyme of an AHL-degrading marine strain of *Bacillus licheniformis* reduces shrimp (*Penaeus indicus*) and common carp (*Cyprinus carpio*) intestinal colonization and mortality by *Vibrio parahaemolyticus* [123,276]. Finally, addition of an AHL-degrading *B. thuringiensis* strain has proved to protect rainbow trout (*Oncorthynchus mykiss*) from *Yersinia ruckeri* infection [277].

Another important application of QS disruption is the prevention of biofouling. Formation of biofilms on ships and in wastewater treatment facilities are in many occasions regulated by QS mechanisms, and they cause significant economic losses [278,279]. Nowadays, different QQ enzymes have been immobilized in nanoparticles, nanofibers, nanotubes, entrapping sheets, and other types of inorganic devices, successfully reducing or preventing biofouling [178,280,281,282,283,284,285,286]. This novel treatment is presented as a promising alternative in the cleaning process of filtering systems in the wastewater treatment plants and in the maintenance of ships, entailing a considerable reduction in the frequency and cost of such processes [69,287].

Last, QSIs and mostly QQ organisms isolated from marine and saline environments could also be used in the future in agriculture since many bacterial phytopathogens that induce economic losses control their virulence or virulence associated functions through QS (Table 1) (reviews: [288,289]). This is the case for instance of *Pectobacterium carotovorum* [186,188,290] (review: [291]), *P. atrosepticum* [179,180], *Erwinia amylovora* [173] (review: [292])*, Burkholderia glumae* (review: [293]), *Ralstonia solanacearum* (review: [294]), and *Agrobacterium tumefaciens* (review: [19]) that regulate motility, plasmid transfer, and the synthesis of macerating exoenzymes, amongst others, through such intercellular communication systems. To date, promising results have been obtained using different compounds or bacterial strains to quench QS-regulated virulence function in *in vivo* assays in plants, for instance, in tomato (*Solanum lycopersicum;* [295,296]) or potato (*Solanum tuberosum*; [70,71,74,115,183,297]). With global changes arising, the world may face a raise of seawater level (review: [298]), generating an increased salinity of underground water and arable areas especially in low lands or fertile river deltas [299,300] even in temperate regions (review: [301]). While researchers and breeders have started to generate important crop cultivars with increased tolerance to salt [302] (review: [303]) the existence of salt tolerant AHL-degrading bacteria may become an asset to control phytopathogens in a context of increasing food demand and increasing world population.

## Figures and Tables

**Table 1:** Virulence-associated QS and QQ/QSI disruptors in proteobacteria of marine and agricultural importance.

Bacterium	QS Signal Molecules		QS-Regulated Phenotypes	References	Possible QS Disruptors		References
	AHLs	Others			QQ Enzymes	QSI Compounds	
**Of marine importance**							
*Aeromonas hydrophila*	C4-HSL, C6-HSL	AI-2	Production of extracellular protease and biofilm formation	[120,121,122]	AiiA lactonase	Vanillin, plant extracts and caffeine	[123,124,125,126,127]
*Aeromonas salmonicida*	C4-HSL, C6-HSL, 3OC6-HSL, C10-HSL	AI-2	Production of extracellular protease	[120,121,128]	-	Sulphur-containing AHL-analogues	[129]
*Aliivibrio fischeri*	3OC6-HSL, C8-HSL	AI-2	Bioluminescence	[130]	-	-	-
*Aliivibrio salmonicida*	C6-HSL, 3OC6-HSL	AI-2	Biolumenescence and biofilm	[32,40]	-	-	-
*Edwarsiella tarda*	C4-HSL, C6-HSL, 3OC6-HSL, C7-HSL	AI-2	Production of extracellular protein	[39,131,132]	Aii20J lactonase	Small peptides	[133,134]
*Vibrio alginolyticus*	3OHC4-HSL, 3OC10-HSL, 3OHC14-HSL	AI-2	Biofilm formation	[42,135]	-	-	-
*Vibrio anguillarum*	C6-HSL, 3OC10-HSL, 3OHC10-HSL	AI-2, CAI-1	Biofilm formation, Production of metalloprotease and pigments	[46]	Aac-like acylase	Furanones; cinnamaldehyde analogs	[41,104,136,137]
*Vibrio campbelli*	3OHC4-HSL	AI-2, CAI-1	Production of metalloprotease, siderophores and chitinase A	[30,44,45,138,139]		Furanones	[140]
*Vibrio corallilyticus*	C4-HSL 3OH,C10-HSL	AI-2	Control of motility, production of hemolysin, caseinase, amylase and alkaline phosphatase	[47,141]	HqiA lactonase, QuiP-like acylase, AiiA lactonase, AttM lactonase	-	[47,105]
*Vibrio harveyi*	3OHC4-HSL	AI-2, CAI-1	Bioluminescence, type III secretion system, extracellular toxin, metalloprotease and siderophore	[48,142,143]	AiiA lactonase	Furanones; 2,6-di-*tert*-butyl-4-methylphenol; cinnamaldehyde analogs; pyrogallol and analogs, AI-2 analogs	[137,140,144,145,146,147,148,149]
*Vibrio mediterranei*	C4-HSL, C6-HSL, 3OHC12-HSL 3OC13-HSL	AI-2	Control of motility, production of DNAse, and chitinase	[22,47]	HqiA lactonase, Aac-like acylase, AiiA lactonase, AttM lactonase	-	[47,104,150]
*Vibrio owensii*	C12-HSL, 3OHC12-HSL	-	Control of motility, production of hemolysin, amylase, DNAse, chitinase and phosphatase	[47]	HqiA lactonase, AiiA lactonase, AttM lactonase	-	[47]
*Vibrio vulnificus*	C4-HSL, 3OC6-HSL 3OHC6-HSL	AI-2	Production of metalloprotease, exoprotease and hemolysin	[36,151]	-	2,6-di-*tert*-butyl-4-methylphenol; cinnamaldehyde analogs	[137,144]
**Of agricultural importance**							
*Agrobacterium tumefaciens*	3OC8-HSL, 3OHC8-HSL		Virulence plasmid conjugation	[152,153,154]	AttM (BlcC) lactonase, AiiB lactonase	Floridoside, betonicine, isethionic acid, thiolactones, dimethyl disulfide, hordenine, estrone	[95,155,156,157,158,159,160]
*Burkholderia glumae*	C6-HSL, C8-HSL	-	Production of the phytotoxin toxoflavin and lipase, biogenesis of flagella, control of internal osmolarity	[161,162,163,164]	AiiA lactonase	AHL- analog J8-C8 (d)	[165,166]
*Dickeya dadantii*	C6-HSL, 3OC6-HSL	-	Partial control of pectate lyase synthesis, control of motility and cell aggregation	[167,168]	AiiA lactonase	-	[169,170]
*Dickeya solani*	C6-HSL (a), C8-HSL	Unknown (Vfm system)	Partial control of the production of macerating exoenzymes	[171,172]	-	-	-
*Erwinia amylovora*	3OC6-HSL, 3OHC6-HSL	AI-2	Possible partial control of virulence	[173,174,175]	-	-	-
*Pantoea stewartii*	3OC6-HSL		Production of exopolysaccharide Bacterial adhesion and biofilm formation	[176,177]	AiiO lactonase	-	[178]
*Pectobacterium atrosepticum*	3OC6-HSL, C8-HSL, 3OC8-HSL, C10-HSL		Production and secretion of macerating exoenzymes, production of harpin, control of motility	[179,180,181,182]	AttM (BlcC) lactonase, AiiB lactonase, AiiA lactonase, QsdA lactonase	*N,N*’-alkylated imidazolium-derivatives	[183,184,185]
*Pectobacterium carotovorum*	C6-HSL, 3OC6-HSL, 3OC8-HSL	AI-2 (b)	Production of macerating exoenzymes and antibiotics	[186,187,188,189,190]	HqiA lactonase, QuiP-like acylase, AiiA lactonase, AhlD lactonase, QsdA lactonase, QlcA lactonase, QsdB amidohydrolase, unidentified oxidoreductase	Furanones, dimethyl disulfide	[70,74,91,103,105,108,112,115,159,191,192]
*Ralstonia solanacearum*	C6-HSL (c), C8-HSL	3OH-PAME	Production of exopolysaccharide I and macerating exoenzymes	[13]	β-hydroxy-palmitate methyl ester hydrolase	-	[116]
*Xanthomonas campestris*	-	DSF (cis-11-methyl-2-dodecenoic acid)	Production of exopolysaccharide and exoenzymes	[16]	Degradation of DSF by unidentified bacterial activities	-	[118]
*Xyllela fastidiosa*	-	Xf-DSF (12-methyl-tetradecanoic acid)	Adhesin production, biofilm stability, insect transmission, production of outer membrane vesicules and attachment to plant vessel cells	[193,194,195]	-	-	-

(a) AHLs are not the main signals regulating virulence; (b) AHLs are the main signals regulating virulence; (c) AHL functions are unknown but AHLs do not regulate virulence; (d) This compound inhibits AHL synthesis and not AHL detection.
